# *KIAA1462*, A Coronary Artery Disease Associated Gene, Is a Candidate Gene for Late Onset Alzheimer Disease in *APOE* Carriers

**DOI:** 10.1371/journal.pone.0082194

**Published:** 2013-12-12

**Authors:** Deborah G. Murdock, Yuki Bradford, Nathalie Schnetz-Boutaud, Ping Mayo, Melissa J. Allen, Laura N. D’Aoust, Xueying Liang, Sabrina L. Mitchell, Stephan Zuchner, Gary W. Small, John R. Gilbert, Margaret A. Pericak-Vance, Jonathan L. Haines

**Affiliations:** 1 Center for Human Genetics Research and Department of Molecular Physiology and Biophysics, Vanderbilt University, Nashville, Tennessee, United States of America; 2 Miami Institute for Human Genomics, Miller School of Medicine, University of Miami, Miami, Florida, United States of America; 3 Semel Institute for Neuroscience and Human Behavior, University of California Los Angeles, Los Angeles, California, United States of America; McGill University Department of Neurology and Neurosurgery, Canada

## Abstract

Alzheimer disease (AD) is a devastating neurodegenerative disease affecting more than five million Americans. In this study, we have used updated genetic linkage data from chromosome 10 in combination with expression data from serial analysis of gene expression to choose a new set of thirteen candidate genes for genetic analysis in late onset Alzheimer disease (LOAD). Results in this study identify the *KIAA1462* locus as a candidate locus for LOAD in *APOE4* carriers. Two genes exist at this locus, *KIAA1462*, a gene associated with coronary artery disease, and “*rokimi”*, encoding an untranslated spliced RNA The genetic architecture at this locus suggests that the gene product important in this association is either *“rokimi”,* or a different isoform of *KIAA1462* than the isoform that is important in cardiovascular disease. Expression data suggests that isoform f of *KIAA1462* is a more attractive candidate for association with LOAD in *APOE4* carriers than *“rokimi*” which had no detectable expression in brain.

## Introduction

Alzheimer disease (AD) is a devastating neurodegenerative disease affecting more than five million Americans [1]–[3]. Symptoms begin late in life and progress over several years ultimately leaving the individual uncommunicative and bedridden. The cause of AD is complex, but the heritable component has been estimated to be as high as 80% [4]. Recent advances have been made in understanding the genetic component of AD, so that much of what we understand about the mechanism of AD we owe to genetics [5]. Investigations of early onset AD identified mutations in *APP* [6], *PSEN1* [7], and *PSEN2* [8], [9] as causative. *APOE* was identified in early candidate gene studies as associated with late onset AD (LOAD) [10], and remains the most replicated association in the 21 genome wide association studies (GWAS) that have been performed to date [5]. In fact, the association of *APOE* with LOAD still explains more of the population attributable risk than all current non-*APOE* GWAS findings together [5], underscoring the genetic complexity in this disease. Over 40 different loci have been highlighted in GWAS as LOAD susceptibility loci; only a handful of those have been confirmed by follow-up [5]. Thus, much of the heritability in LOAD remains unexplained.

The association between coronary vascular disease (CVD) and LOAD remains unclear. It has been theorized that atherosclerosis resulting in compromised blood flow to the brain and subsequent oxidative stress and inflammation could contribute to the risk for LOAD [11]. *APOE* has also been linked to CVD, although this association is controversial [12], [13]. It appears, however, that the contribution of *APOE* to AD pathology is not through enhanced CVD, but through more direct effects on amyloid beta processing and neurotoxicity [14].

In this study, we have used updated genetic linkage data from chromosome 10 in combination with expression data from serial analysis of gene expression to choose a new set of thirteen candidate genes for genetic analysis in LOAD. Chromosome 10 has long been of interest in LOAD genetics based on linkage studies [15]–[22]. Results in this study identify the *KIAA1462* locus as a candidate locus for LOAD in *APOE4* carriers. The likelihood that this gene is a candidate gene for LOAD in *APOE* carriers is discussed.

## Materials and Methods

### Study Populations

All individuals included in this study were Caucasian late-onset AD (LOAD) participants (minimum age at onset (AAO) = 60 years) and related unaffected relatives. LOAD was diagnosed according to the NINCDS­ADRDA criteria [23]. All unaffected individuals had results within the normal range in the Mini-Mental State Exam (MMSE) or Modified Mini-Mental State Exam (3 MS). Families were chosen by the presence of two or more affected individuals. Samples from all affected and at least one unaffected first degree relative were collected, resulting in an increased number of affected over unaffected individuals in this study. The overall data set of 441 families contains 1001 affected and 352 unaffected individuals (see Table 1 for details). The number of affected women is also more than the number of affected men, reflecting the increased incidence of LOAD in females [24] and the general tendency for women to participate in research at a higher rate then men. Samples were ascertained by the following centers: the National Cell Repository for Alzheimer’s Disease at Indiana University (NCRAD); the Collaborative Alzheimer Project (CAP), including the University of Miami, Vanderbilt University, the University of California at Los Angeles; and the National Institute of Mental Health repository (NIMH). Written consent was obtained from all participants in agreement with protocols approved by the institutional review board for the CAP participants at the University of Miami, Vanderbilt University, and the University of California at Los Angeles. Following informed consent, blood samples were collected from each individual and genomic DNA was extracted using the Puregene system (Gentra Systems, Minneapolis, MN). Extracted DNA was obtained from the NCRAD and NIMH repositories.

**Table 1:** AD family data details.

	ALL*	NCRAD*	NIMH*	CAP*
Number of Families	441	85	289	67
Number of Individuals	1354	250	877	227
Number of Affected Ind.	1001	189	644	168
Sex				
Female	708	133	477	98
Male	293	56	167	70
Number of Unaffected Ind.	352	61	232	59
Sex				
Female	208	32	144	32
Male	144	29	88	27
Number of Unknown Ind.	1	0	1	
Sex				
Female	0	0	0	0
Male	1	0	1	0
Number of Families with:				
0 affected	1	0	1	0
1 affected	7	0	7	0
2 affected	336	71	219	46
3 affected	73	11	50	12
4 affected	20	2	11	7
5 affected	2	0	1	1
6 affected	1	1	0	0
7 affected	1	0	0	1
8 affected	0	0	0	0
Number of Families	441	85	289	67

**ALL** column lists the combined totals for all ascertainment sites. Samples were ascertained by the following centers: the NCRAD repository at Indiana University (**NCRAD**); the Collaborative Alzheimer Project (**CAP**), including the University of Miami and Vanderbilt University and University of California at Los Angeles; and the National Institute of Mental Health repository (**NIMH**). The

### Gene Selection

The Serial Analysis of Gene Expression (SAGE) method was used to compare the gene expression levels in the brain tissue from LOAD patients and controls as described elsewhere [25], [26]. Candidate genes for analysis in this study were chosen by the convergence of their differential expression data in LOAD brain compared to control brain, and their position under the LOAD linkage peaks as shown in previous linkage studies.

### SNP Selection and Genotyping

Tagging SNPs were selected using LDSelect with an *r* ^2^ threshold of 0.8 in the CEU subject data of HapMap Release 21 of phase II of the National Center for Biotechnology Information build 35 assembly. CEU subjects were Utah residents of northern and western European ancestry from the Centre d’Etude du Polymorphisme Humain. The minor allele frequency threshold was 0.05, as exceeded by any Caucasian study population of dbSNP, including CEU.

A total of 384 SNPs on chr10 were genotyped with the use of the midthroughput Sequenom genotyping platform, based on a single-base primer extension reaction coupled with mass spectrometry. The assays were designed using Sequenom SpectroDESIGNER software. Genomic DNA (5 ng) was amplified following the manufacturer recommendations (Sequenom). Single primer extension over the SNP was carried out in a final concentration of 1.25 µM of the extension primer. The extension step followed the manufacturer procedure. The reaction was then desalted by addition of 6 mg of resin followed by 15 min mixing and centrifugation (3000 rpm) to settle the contents of the plate. The extension product was then spotted onto a 384 well spectroCHIP before being flown in the MALDI-TOF mass spectrometer. Data was collected, real time, using SpectroTYPER Analyzer, SpectraAQUIRE and SpectroCALLER (Sequenom). DNA samples from cases and controls were randomly sorted, and duplicate samples were implemented across plates for genotyping quality control.

### Statistical Methods

#### Association analysis in family data set

The allelic association analyses were conducted using the association in the presence of linkage (APL) program [27] and the pedigree disequilibrium test (PDT) [28]. These methods provide valid and robust tests for allelic association in trios and extended families. The Genotype-PDT (GenoPDT) tested genotypic association to the risk of LOAD [29]. Genotype efficiency, Hardy-Weinberg Equilibrium and linkage disequilibrium were checked using Haploview [30]. Linkage analysis was conducted on families using two-point heterogeneity LOD scores (HLOD) calculated using FASTLINK and HOMOG [31]. Both recessive and dominant models with disease allele frequencies of 0.01 and 0.001, respectively, were analyzed. This approach is robust for detecting linkage signals when the underlying model is unknown or complex [32].

### Reverse Transcriptase PCR and Real Time Quantitative PCR

Frozen superior frontal cortex samples were obtained from the Harvard Brain Tissue Resource Center from four control and four LOAD brains. RNA was isolated from these samples using TRIzol reagent (Invitrogen) and converted to first strand cDNA using the SuperScript® III First-Strand Synthesis System for RT-PCR (Invitrogen). Oligo(dT), random hexamers, and a gene specific primer were all used separately to create first strand synthesis for the *rokimi* putative transcript, while oligo(dT) was used as a first strand synthesis primer for the *KIAA1462* mRNA quantitation. TaqMan probes specific for the junction between exons 2 and 3 (Hs1584907_m1), between exons 3 and 4 (Hs1584907_m1), and the 3′UTR (6835–6843 of NM_020848) were used for quantitative real time PCR. Relative levels of the *KIAA1462* transcript were measured with quantitative real time PCR on an ABI 7900HT Fast Real Time PCR System using Taqman Gene Expression Assays. Absolute quantitation was performed using standard curves based on cDNAs cloned into the pCR4-TOPO vector using the TOPO-TA cloning kit (Invitrogen). Endogenous control assays to housekeeping gene *GAPDH* or *ACTB* were run in triplicate. The presence or absence of the *rokimi* transcript in brain was determined by the presence/absence of an RT-PCR product of the correct size. Two sets of primers were used, primer set 1 (5′-ctcctgcccttctcccatc-3′ roki1.for and 5′-ggcacgatcttggctcat-3′ roki1.rev) primers set 2 (5′-CACTCCTAGGCGGGGCTCCT-3′ roki2.for and 5′-TGCGTACCTCACCGAGGTTTC-3′ roki2.rev).

## Results

### Genomic Convergence Identified Genes and Genotyped SNPs

To identify new candidate genes for Alzheimer disease, linkage data from chromosome 10 was combined with expression data from SAGE analysis. Genes that showed significant differential expression in AD brain in at least two SAGE comparisons [33] and were under previously identified linkage peaks [16], [18], [34]–[36] were chosen for further study. Thirteen genes were selected for detailed genetic analysis, and all of these genes have evidence for expression in brain in the Unigene database. Tagging SNPs were chosen from both intronic and exonic regions of the genes, except for *PRKG1* where size necessitated limiting SNPs to the exonic regions. The gene description, size, and number of genotyped SNPs are summarized in Table 2.

**Table 2:** Candidate Genes on Chromoesome 10 Identified by Genomic Convergence.

Gene Name	UniGene	Expression Ratio*	start position	Size (kb)	# of SNPs genotyped
DNMBP	Hs.500771	−14.4	101625324	134342	12
TMEM10	Hs.12449	−6.3	98092968	16081	13
SORBS1	Hs.38621	1.5	97061520	249605	102
HELLS	Hs.546260	1.7	96295563	56282	6
LOC387700	Hs.530338	1.6	91180035	105256	32
IFIT3	Hs.549041	1.7	91082265	8023	3
FAS	Hs.244139	1.6	90740267	25254	30
C10orf58	Hs.500333	1.7	82167953	6188	7
PRKG1	Hs.2689	1.7	52504298	1220982	92
C10orf72	Hs.522928	2	49893191	3018	21
CUL2	Hs.82919	2.2	35338813	80487	5
EPC1	Hs.167805	1.7	32610792	65360	14
KIAA1462	Hs.533953	3.1	30344113	13294	47

[33]. Expression ratio is from SAGE data

### Association Analysis and Real Time Quantitative PCR of *KIAA1462*

**Figure 1:**
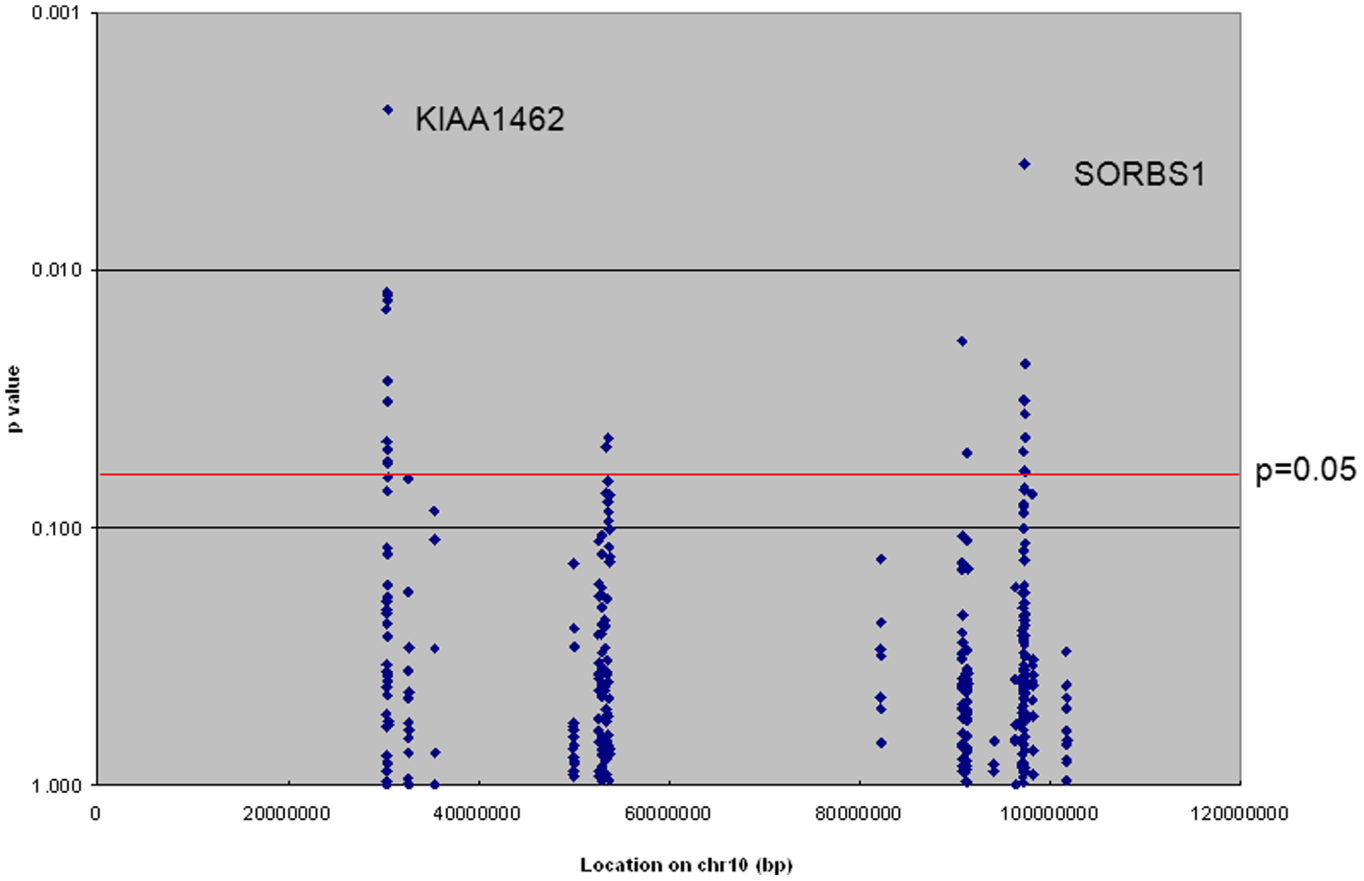
Allelic association test of candidate gene SNPs in the overall data set. *KIAA1462* and *SORBS1* were associated with LOAD at a p value<.01.

**Figure 2:**
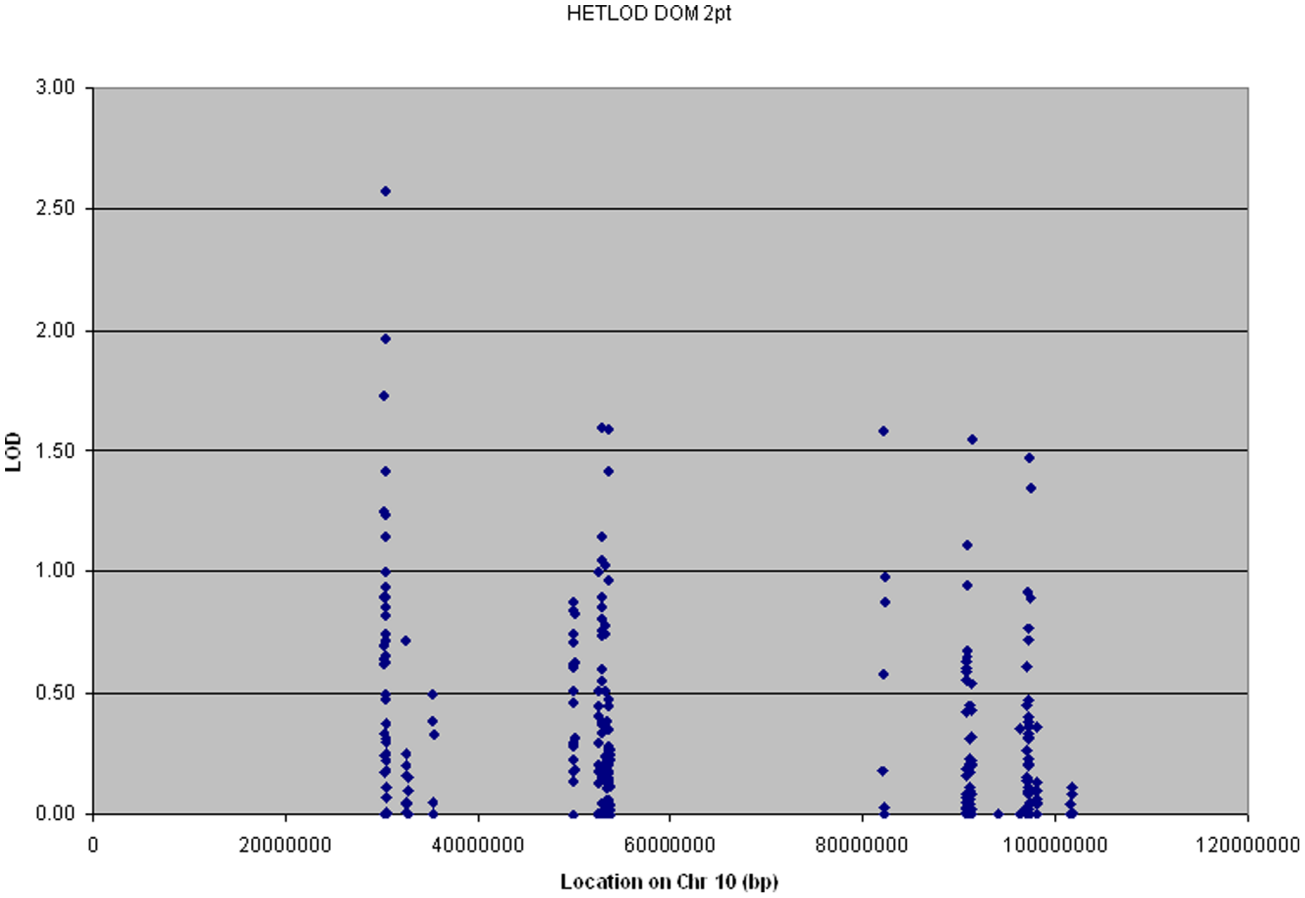
Two point heterogeneity LOD scores of candidate gene SNPs in the overall data set.

The allelic association of 384 genotyped SNPs in thirteen genes was analyzed in the overall data set of 441 families containing 1001 affected and 352 unaffected individuals (Table 1). The Genotype-PDT (GenoPDT) results are presented in Figure 1 and the linkage analysis results using two-point heterogeneity LOD scores (HLOD) are presented in Figure 2. Fourteen SNPs in six genes (*KIAA1462*; MIM# 6, *CUL2*; MIM#603135, *PRKG1*; MIM# 176894, *FAS*; MIM#134637, *SLC16A12*; MIM#611910, and *SORBS1*; MIM#605264) showed association with AD at nominal p<0.05. Two of those SNPS, rs2488024 in *KIAA1462* and rs10823056 in *PRKG1*, also had calculated two point LOD scores greater than 1.0.

### The Association of *KIAA1462* Locus SNPs and LOAD is Restricted to *APOE* Carriers

GWAS studies have identified *KIAA1462* as a novel locus for coronary artery disease [37], [38]. The association between non-stroke cardiovascular disease (CVD) and Alzheimer’s disease has been debated, but a recent study reports that CVD increases risk of AD only in carriers of the *APOE4* allele (Hazard Ratio 2.39, 95% confidence interval) [39]. From these studies, a model is suggested in which variation in *KIAA1462* increases the risk of coronary artery disease, which in turn increases the risk of LOAD only in *APOE4* carriers. To test this model, the genetic analysis of the association between SNPs in *KIAA1462* and LOAD was performed again after stratifying the families by *APOE* status. The *APOE* status of a family was defined as positive if more than 50% of the family members had the *APOE4* allele. By this definition, 279 *APOE* positive families and 162 *APOE* negative families were analyzed. When the analysis is stratified by *APOE* status, the association between *KIAA1462* and LOAD is significant almost exclusively in the *APOE* positive group (Table 3).

**Table 3:** Linkage of SNPs on KIAA1462/rokimi region of chr10 and LOAD unstratified (all families) and stratified by APOE status.

	All families				APOE positive families				APOE negative families			
	HETLOD	HETLOD	PDT		MLOD	MLOD			MLOD	MLOD		
RS#	dom	REC 2pt	SUM	FBAT	dom	rec	PDTsum	FBAT	dom	rec	PDTsum	FBAT
RS2256283	0.33	0.10	1.000	0.997	0.24	0.05	0.739	0.879	0.10	0.05	0.587	0.850
RS3006544	0.00	0.00	0.665	0.968	0.00	0.00	0.962	0.351	0.00	0.02	0.549	0.255
RS12764479	0.00	0.00	0.677	0.878	0.00	0.00	0.232	0.359	0.08	0.14	0.091	0.073
RS7917431	0.24	0.25	0.483	0.528	0.02	0.03	0.652	0.453	0.41	0.45	0.587	0.891
RS1774240	0.90	0.76	0.213	0.207	0.43	0.28	0.214	0.341	0.68	0.57	0.685	0.390
RS1774243	0.64	0.84	0.212	0.445	0.76	**1.02**	0.475	0.854	0.02	0.04	0.283	0.266
RS1418278	1.73	1.66	0.515	0.360	**1.11**	0.89	0.291	0.160	0.69	0.79	0.775	0.870
RS1063205	0.70	0.66	0.294	0.193	0.28	0.34	0.351	0.261	0.48	0.32	0.602	0.394
RS3739998	0.86	0.70	0.308	0.265	0.69	0.56	0.390	0.494	0.20	0.15	0.580	0.295
RS2185724	0.11	0.10	0.572	0.416	0.04	0.08	0.513	0.464	0.07	0.02	0.904	0.733
RS9337951	1.00	0.69	0.362	0.375	0.64	0.36	0.317	0.390	0.36	0.38	0.874	0.543
RS2487928	1.24	0.95	0.077	0.072	**1.02**	0.77	0.151	0.152	0.26	0.21	0.299	0.154
RS1342150	0.48	0.29	0.077	0.046	0.17	0.06	0.236	0.317	0.37	0.30	0.186	**0.032**
RS2478833	0.90	0.68	0.020	0.012	0.26	0.10	0.100	**0.021**	0.74	**1.07**	0.094	0.156
RS2505087	1.42	1.30	0.063	0.027	**1.39**	**1.01**	0.155	**0.037**	0.15	0.43	0.209	0.180
RS1122730	1.96	1.07	0.235	0.235	**1.67**	0.76	0.581	0.430	0.37	0.32	0.203	0.257
RS2255515	0.18	0.06	0.109	0.119	0.19	0.02	0.283	0.224	0.02	0.05	0.128	0.248
RS6481654	0.18	0.16	0.050	0.235	0.38	0.32	0.113	0.231	0.00	0.00	0.245	0.446
RS4749527	0.37	0.30	0.286	0.395	**1.31**	**1.13**	0.298	0.292	0.00	0.00	0.752	0.783
RS2505127	0.30	0.15	0.021	0.056	0.15	0.00	0.064	0.087	0.24	0.44	0.131	0.281
RS3847404	0.25	0.13	0.088	0.064	0.57	0.35	0.201	0.095	0.00	0.00	0.234	0.237
RS2505126	0.94	0.60	0.005	0.013	**1.07**	0.68	**0.017**	**0.015**	0.03	0.01	0.129	0.173
RS12220246	0.00	2.91	0.445	0.032	0.09	**2.02**	0.589	**0.033**	0.00	0.93	0.582	**0.030**
RS2488024	1.00	0.65	0.000	0.002	0.77	0.48	**0.004**	**0.009**	0.23	0.18	**0.030**	**0.041**
RS11007904	0.72	0.56	0.096	0.167	**1.27**	**1.16**	0.098	0.087	0.00	0.00	0.630	0.750
RS1887317	1.15	1.18	0.100	0.265	0.58	0.51	0.065	0.146	0.62	0.73	0.889	0.921
RS10826765	0.22	0.14	0.029	0.056	0.46	0.36	0.146	0.100	0.00	0.00	0.079	0.212

### The Association of the KIAA1462 Locus with LOAD in a Publicly Available Genome-wide Dataset

The region of interest was examined for association with LOAD using the dataset assembled by the ADGC (stage 1, 8,309 individuals with LOAD (cases) and 7,366 cognitively normal elders (CNEs) as controls) from eight cohorts and a ninth newly assembled cohort from the 29 National Institute on Aging (NIA)-funded Alzheimer Disease Centers (ADCs) [40]. No significant association with LOAD was seen at this locus in this dataset. Although these data might suggest that KIAA1462 is not associated with LOAD, it is also possible that an association would only be detected after stratification by APOE status or presence of cardiovascular disease in this dataset. Thus a large scale GWAS study cannot exclude that this gene is still important in a subset of LOAD.

### Analysis of the Genomic Structure at the *KIAA1462* Locus Identifies another Putative Gene

**Figure 3:**
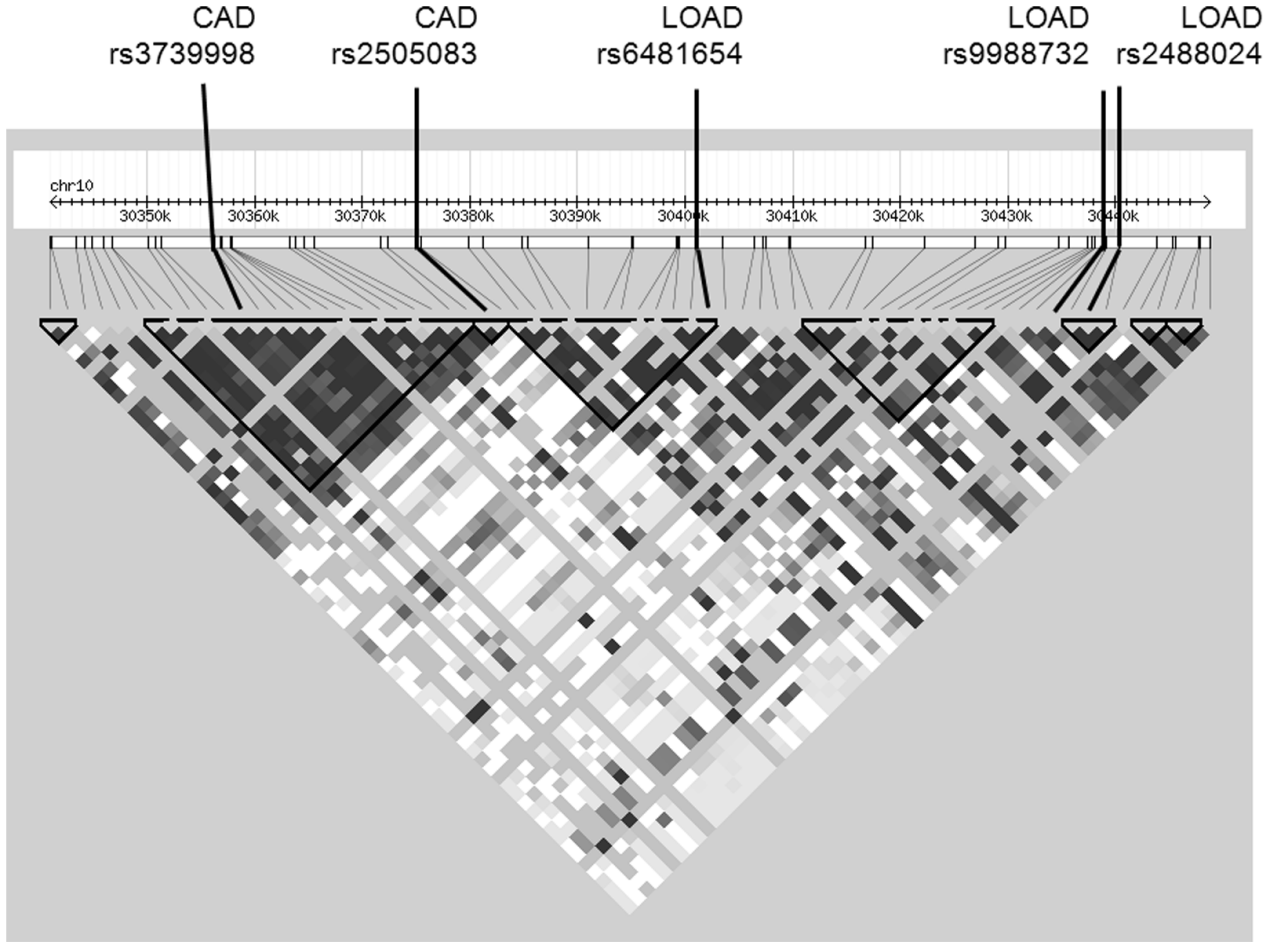
Linkage disequilibrium plot of SNPs associated with LOAD and CAD in *KIAA1462* region.

**Figure 4:**
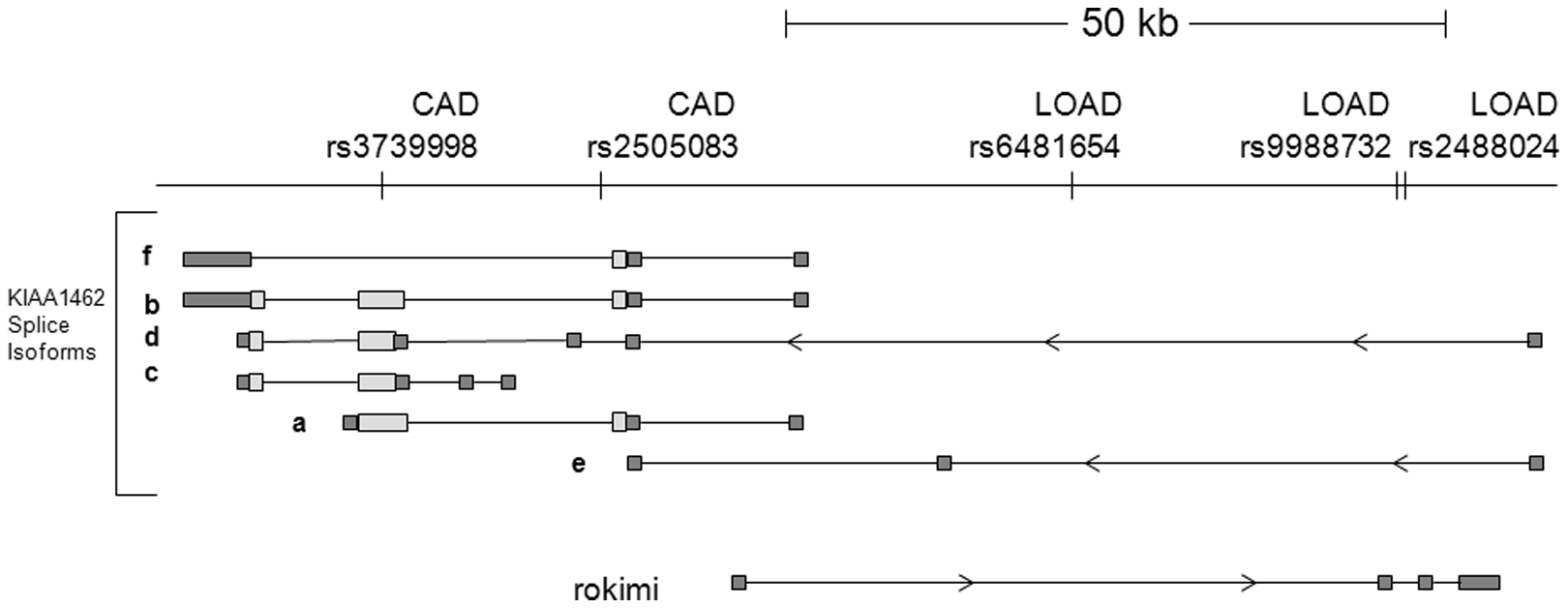
CAD and LOAD associated SNPs mapped to intron/exon map of expressed genes. SNPs associated with CAD and LOAD are shown on the top line. Isoforms of *KIAA1462* and *“rokimi*” are shown in relation underneath. Direction of transcription is symbolized by arrows. Exons are shown as boxes, and introns as lines. Putative protein coding regions of exons are shown as lighter colored boxes, and noncoding regions as darker boxes.

While three SNPS in the *KIAA1462* locus showed nominal association to AD in a previous study, none of these associations survived multiple testing correction [41]. More recently, other SNPs in the locus were discovered in two GWAS to be strongly associated with coronary artery disease (CAD) (p = 8.78×10^−6^) [42], [37]. When Haploview [30] was used to analyze linkage disequilibrium, the groups of SNPs associated with CAD and LOAD appear to cluster in two different LD blocks, suggesting that they are inherited separately (Figure 3). In addition, when the SNPs from the two studies were overlaid on the genomic structure of the locus using the UCSC genome browser (February 2009 human reference sequence (GRCh37)) [43], the SNPs associated with CAD are more distal than the SNPs associated with LOAD (Figure 4). The CAD SNPs are clustered over the protein coding exons of *KIAA1462*, while the LOAD SNPs are clustered proximal to the protein coding exons.

*KIAA1462* has seven predicted splice isoforms, four of which have protein coding exons (Aceview) (Figure 4). The isoform b structure, which contains all three coding exons, is conserved in mouse, and the predicted protein is expressed in mouse [42]. The SNPs associated with CAD are more distal than the SNPs associated with LOAD, which are clustered upstream of the predicted start of transcription of *KIAA1462* isoforms a, b, c, and f, and in the introns of isoforms d and e. Another gene, designated “*rokimi*” in Aceview, was recently identified as a noncoding spliced RNA completely internal to the *KIAA1462* gene, but on the opposite strand of DNA. The sequence of the *“rokimi”* gene is defined by two GenBank accessions from one cDNA clone from synovial membrane tissue from a patient with rheumatoid arthritis. The SNPs associated with LOAD are also within introns of the “*rokimi”* gene.

### Analysis of Expression Levels of *KIAA1462* and *“rokimi”*

The location of the SNPs associated with LOAD suggests that they might alter the transcription of *KIAA1462* or *“rokimi”.* The expression of *KIAA1462* was investigated in brain of LOAD and control individuals using real time quantitative PCR. *KIAA1462* mRNA was measured in brain samples taken from 14 control and 14 LOAD individuals (Table 4) using TaqMan based real time quantitative PCR. Three TaqMan probes were used individually, one specific for the junction between exons 2 and 3 of isoform b, one specific for the junction between exons 3 and 4 of isoform b, and one in the 3′ untranslated region of isoform b. These probes were designed to measure expression of isoforms a, b, c, and d. *KIAA1462* was not significantly differentially expressed in AD brains as compared to controls (data not shown) using any of these probes. In addition, *KIAA1462* was not differentially expressed in the brain based on any APOE genotype (data not shown).

**Table 4:** Origin of Brain Samples for RNA Purification.

Sample	Gender	BRAAK Stage	Age of Onset	Age at Exam	ApoE Allele 1	ApoE Allele 2	Origin
C01	M			68	3	3	HBTRC
C02	M			66	3	4	HBTRC
C03	M			67	2	3	HBTRC
C04	M			76	3	3	HBTRC
C05	M			88	3	3	HBTRC
C06	F			95	3	3	HBTRC
C07	M			78	3	3	HBTRC
C08	M			78	3	3	HBTRC
C09	M			67	3	3	HBTRC
C10	F			78	3	3	HBTRC
C11	F			84			Xu, et.al. 2007
C12	M			75			Xu, et.al. 2007
C13	M			74			Xu, et.al. 2007
C14	F			79			Xu, et.al. 2007
AD1	M	IV	69	77	4	4	HBTRC
AD2	M	III	76	84	3	3	HBTRC
AD3	M	V	73	81	4	4	HBTRC
AD4	F	V	68	76	4	4	HBTRC
AD5	M	IV	69	75	4	4	HBTRC
AD6	M	VI	71	75	3	4	HBTRC
AD7	F	VI	75	81	3	4	HBTRC
AD8	F	V	75	86	3	3	HBTRC
AD9	F	VI	81	89	3	3	HBTRC
AD10	F	V	85	91	3	3	HBTRC
AD11	M	VI		70			Xu, et.al. 2007
AD12	F	VI		79			Xu, et.al. 2007
AD13	M	VI		77			Xu, et.al. 2007
AD14	F	VI		87			Xu, et.al. 2007

The presence of expression of the *rokimi* gene was examined using RT-PCR. RNA from brain and HeLa cell lines was used to perform RT-PCR with two sets of *rokimi* primers. Because *rokimi* appears to be a noncoding RNA and it was unclear if the message would be polyadenylated or not, in addition to oligo(dT) primers for first strand synthesis, random hexamers and a gene specific primer were also used. None of these experimental conditions yielded a PCR product, suggesting that *rokimi* is not expressed in brain tissue or HeLa cells. We have also examined the KIAA1462/rokimi locus for QTLs using both the eQTL Browser [44] and Aperture, an online tool designed by the William Bush laboratory (http://gwar.mc.vanderbilt.edu/aperture) to provide LocusZoom-style plots for over 28 million eQTL associations. No significant eQTLs were found at this locus.

## Discussion

Ours is not the first genomic convergence study to investigate *KIAA1462* in relation to AD and find similar results. Chapuis et al. selected genes from areas of genetic interest in LOAD, analyzed their expression in LOAD brain compared to controls via custom expression microarrays, and found that *KIAA1462* was upregulated approximately 2.5 fold in AD brain [41]. Follow-up examination of eighteen SNPs in *KIAA1462* showed that three were associated with AD, including rs2488024, the most strongly associated SNP in our study, and five were associated with age on onset of AD. The significance of the association of these SNPs did not, however, survive multiple correction testing [41].

Polymorphisms in *KIAA1462* have also been associated with other diseases and phenotypes. *KIAA1462* was one of six loci associated with variation in human recombination rates [45]. Specifically rs2505089 in *KIAA1462* was found by GWAS to be associated with maternal recombination rate with a combined p value = 4.42×10^−7^. In addition, recent reports link *KIAA1462* to CAD with genome wide significance scores [37], [38]. The first study showed association of rs3739998, a nonsynonymous SNP in *KIAA1462*, with CAD in the German MI Family Study with a combined p value = 1.27×10^−11^ [38]. The second study, a meta-analysis of four combined GWAS with subjects from both European and South Asian background, revealed five major loci associated with CAD, including *KIAA1462* (p value = 3.87×10(−8)) [37]. The function of *KIAA1462* is unknown, but the protein encoded by isoform b is localized to endothelial cell-cell junctions, and this localization is VE-cadherin dependent [42].

Our results support those from another study examining chromosome 10 variation in LOAD [41]. Taken together, these results bolster the idea that *KIAA1462* might play some role in the development of LOAD, although the role would likely be quite small. We can not rule out, however, that this locus is not associated with LOAD, considering that the data associating the locus with late onset AD is modest. The role of *KIAA1462* in the genetics of CAD, however, seems much clearer from recent GWAS [37], [38]. The association between non-stroke cardiovascular disease (CVD) and Alzheimer’s disease has been debated, but a recent study reports that CVD increases risk of AD only in carriers of the *APOE*4 allele (Hazard Ratio 2.39, 95% confidence interval) [39]. The present study shows that *KIAA1462*, which is associated with CVD, is associated with *APOE* positive LOAD. The discovery of a genetic modifier of CVD that is associated with the *APOE4* class of LOAD might explain some of the conflicting results in the literature regarding CVD and LOAD. These results suggest a model in which underlying CAD increases the risk of LOAD only in *APOE4* carriers.

Several different lines of evidence suggest that variation in two different gene products at this locus may be responsible for the association with CAD and LOAD respectively. The CAD associated SNP rs3739998, which is a nonsynonymous change in exon 3 of isoform b of *KIAA1462* that changes a serine to a threonine, is associated with CAD with a p value of 1.27×10^−11^, but is not significantly associated with LOAD in our study. Secondly, the genomic arrangement of the SNPs associated with CAD and LOAD suggests that they are in two different linkage disequilibrium blocks. The more proximal nature of the LOAD associated SNPs suggest that the gene product important in this association is either *“rokimi”,* or a different isoform of *KIAA1462* than the isoform that is important in CVD. Since expression of *“rokimi”* was not detected in brain, it is unlikely that *“rokimi”* is the gene of interest in LOAD. We also did not see any differential expression of *KIAA1462* in LOAD brain, but this could be a result of the methods used. With the three different TaqMan probes used, we would have observed a difference in expression of isoforms a, b, c, and d. Isoform e was not recognized by these probes, and isoform f has alternative polyadenylation sites, therefore some forms of isoform f may not have been detected by these TaqMan assays. Isoform e is represented by one Genbank entry from skeletal muscle. Isoform f is represented by 175 Genbank entries from many tissues, including multiple entries from brain, making isoform f a more attractive candidate for association with LOAD.
